# A novel *ex vivo* perfusion-based mandibular pig model for dental product testing and training

**DOI:** 10.1186/s12903-023-02794-6

**Published:** 2023-02-23

**Authors:** Machteld van Erk, Roger M. L. M. Lomme, Edwin A. Roozen, Bart A. J. A. van Oirschot, Harry van Goor

**Affiliations:** 1 Department of Surgery (Route 618), Radboud University Medical Centre, PO Box 9101, 6500 HB Nijmegen, The Netherlands; 2 Department of Dentistry – Regenerative Biomaterials, Radboud University Medical Centre, Philips van Leydenlaan 25, 6525 EX Nijmegen, The Netherlands

**Keywords:** Biomaterials, Guided tissue regeneration, Surgical techniques, Wound healing, Dental model, Perfusion

## Abstract

**Background:**

A translational *ex vivo* perfusion-based mandibular pig model was developed as an alternative to animal experiments, for initial assessment of biomaterials in dental and maxillofacial surgery and training. This study aimed to assess the face and content validity of the novel perfusion-based model.

**Methods:**

Cadaveric porcine heads were connected to an organ assist perfusion device for blood circulation and tissue oxygenation. Dental professionals and dental trainees performed a surgical procedure on the mandibula resembling a submandibular extraoral incision to create bone defects. The bone defects were filled and covered with a commercial barrier membrane. All participants completed a questionnaire using a 5-point Likert scale to assess the face and content validity of the model. Validation data between the two groups of participants were compared with Mann–Whitney U test.

**Results:**

Ten dental professionals and seven trainees evaluated the model for face and content validity. Participants reported model realism, with a mean face validity score of 3.9 ± 1.0 and a content validity of 4.1 ± 0.8. No significant differences were found for overall face and content validity between experts and trainees.

**Conclusion:**

We established face and content validity in a novel perfusion-based mandibular surgery model. This model can be used as an alternative for animal studies evaluating new biomaterials and related dental and maxillofacial surgical procedural training.

## Background

Due to the growing and aging world population, the numbers of oral and maxillofacial procedures and associated products are rising [1–3]. Before new products are allowed to be used in patients, their safety and effectiveness are comprehensively evaluated in in vitro and in vivo studies. A major disadvantage of in vitro studies is that translational value is often limited due to absence of composed tissues and body fluids. In vivo studies are therefore generally used to investigate the initial performance of a new product, and to evaluate long term safety and biological processes. In vivo studies are currently golden standard for final-stage product assessments, however, there is evidence that appropriate *ex vivo* models might replace early animal experiments, particularly for the assessment of initial product performance [4–6]. Benefit of an *ex vivo* model is the reduction in the use of living laboratory animals, which is in line with the 3R philosophy—reduction, refinement, replacement—which encourages researchers to search for alternatives to animal experiments [7].

In order to develop and use new clinically relevant *ex vivo* models for research, a few challenges has to be tackled including the lack of biological factors and parameters (*e.g.* tissue fluids and body temperature). With this in mind, we developed a novel *ex vivo* model for bone adhesive barrier membrane assessment which includes a range of the most important biological parameters for product assessment. This model consists of a cadaveric pig head, which closely simulates the human physiology [8]. A pulsatile blood flow was added to the model, allowing to test the prototype samples on perfused bone tissue. In addition, the pulsatile blood flow has effect on parameters such as pH, temperature, elasticity and fluid content of the (surrounding) tissues. These parameters may alter material characteristics and handling, and affect the performance of the materials after application or implantation. Furthermore, the blood flow affects the surgical procedure to apply the biomaterial, with haemorrhage and means to control a bleeding. A realistic model without the use of living laboratory animals can be a promising tool for initial product evaluation, but also to train surgical procedures without a research intent.

To assess the potential of this novel *ex vivo* model for initial product evaluation and training, the model needs to be validated according to relevant clinical criteria. This study aims to evaluate two types of validity related to our model: (1) face validity, by examining to what extent the model is capable in simulating a clinical situation, and (2) content validity, by examining the ability and sensitivity of the model to assess the dental and maxillofacial surgical procedure and product application. When face and content validity can be confirmed, this model may impact the use of animals in biomaterial research and corresponding training of surgical procedures and techniques.

## Materials and methods

### Animal tissue acquisition

Fresh porcine cadaveric heads and 10L of whole blood were obtained from the slaughterhouse on the day of an experiment. All animals were slaughtered for human consumption. Protocols were in accordance with EC regulations 1069/2009 regarding the use of slaughterhouse animal material for diagnosis and research, and approved by the animal ethical committee of the Radboud University Medical Centre, the Netherlands (project license AVD10300202010866, protocol 2020-0016). For each experiment, fresh mixed (several pigs) porcine blood was collected by exsanguination into a container with 5000 i.u. heparin per litre of blood for reperfusion. The porcine head was stored in an expanded polystyrene box for transport to the laboratory. Total warm ischemia time was 90–120 min.

### Mandibular model

**Fig. 1:**
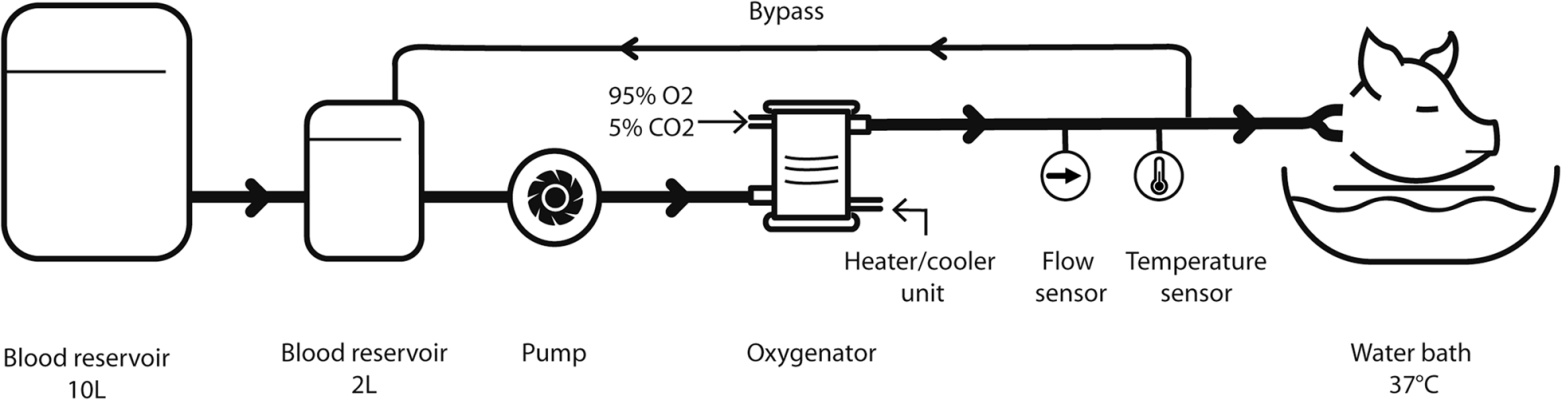
Schematic overview of perfusion system with the connected cadaveric porcine head

In the laboratory (in the central animal facility of the Radboud University Medical Centre, Nijmegen, the Netherlands), porcine heads were connected to an extracorporeal organ perfusion system, the ECOPS (Organ Assist, Groningen, the Netherlands). This device consists of an automatic centrifugal pump system, providing the arterial flow and pressure, a (de-)oxygenator, temperature probes and flow probe (Fig. 1). The blood was oxygenated using a mixture of 95% oxygen and 5% carbon dioxide and heated to 37 °C via the heater/cooler unit. The flow controlled blood pressure was kept stable between 60 and 80 mm Hg and was regulated by (further) opening or closing the bypass. Blood flow was initially set at approximately 150 ml/min [range 150–300 ml/min] and could be adapted via the pump to create stable pressures. Variations between experiments were mainly due to anatomical variations in the heads (*i.e*. large vasculature).

The perfusion system was primed with the blood to stabilize the hemodynamic parameters and to warm the blood to 37 °C. Thereafter, cannulation of both common carotid arteries was performed and major veins at the back of the head were clamped to limit blood loss in the system. A water bath underneath the porcine head was filled with water at 37 °C to keep the tissues warm. After a stabilization time of approximately 30 min, one side of the head was heated (> 30 °C) and the surgical procedure was started at the heated side of the head. After the procedure, the head was turned to continue on the other side of the jaw that was meanwhile heated.

### Study participants

Dentists, dental trainees and oral maxillofacial (OMF) surgeons were recruited to validate the *ex vivo* model. A waiver for ethical approval was obtained from the ethical review board of the Radboud University Medical Centre Nijmegen according to the Dutch Medical Research Involving Human Subjects Act after reviewing the research protocol (Article 3, WMO act). The study was performed in accordance with the Declaration of Helsinki. A minimum of one and a maximum of three persons participated in the experiment simultaneously. Participants were divided based on (oral) surgical experience; trainee (postgraduate year 5 or 6 dental students, having finished a basic maxillofacial surgery teaching module) or expert (dentists or OMF surgeons). This division was made to investigate if the level of experience affects model assessment and to obtain a first impression of usability of the model for skills training in oral surgery treatment. Since the *ex vivo* perfusion-based model is a new model, no data regarding validity scores were present. Therefore, no data for a power calculation was available. We expected to find reliable and possibly significant results with a sample size of ≥ 6, based on previous experience*.*

### Study design and questionnaire

**Fig. 2:**
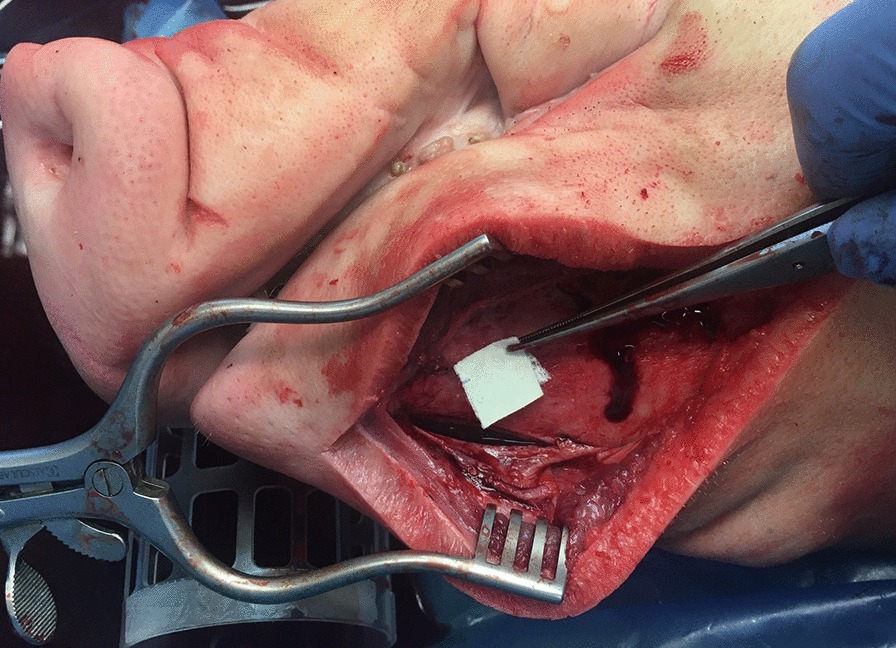
Extraoral submandibular approach on the perfusion-based model which included incision of the soft tissues and elevation of the periosteum. A hole was drilled in the mandibular bone tissue, filled with bone granules and a commercial barrier membrane is applied over the bone defect

Participants received written and oral information about the model and procedure. Thereafter they performed a series of tasks consisting of an extraoral submandibular skin incision, elevating the periosteum, drilling a bone defect in the mandible with a dental implant drill (ø3.35 mm), filling the defect with a bone substitute (Bio-Oss^®^, GeistlichPharma AG, Wolhusen, Switzerland or CreOss™ xenogain, Nobel Biocare, Zürich-Flughafen, Switzerland) and application of a commercial barrier membrane (BioGide^®^, GeistlichPharma AG, Wolhusen, Switzerland) over the bone defect (Fig. 2). After completing all tasks, participants assessed the mandibular pig model using a 5-point Likert scale across a 12-item validation questionnaire (Table 1). Questions were adapted from a previous questionnaire for anatomical and surgical simulation models assessing face and content validity [9]. Face validity was assessed with questions 1–7 and content validity with questions 8–12. Of these content validity questions, questions 8 and 9 concerned task specific content validity while questions 10–12 concerned global content validity. The questionnaire was tested on reliability and uniform text interpretation by a panel of ten medical and dental researchers prior to the experiments. Additional comments on the model and questionnaire by the participants during the experiment were noted by the first author (MvE). Data regarding demographics and years of supervised (trainee) or unsupervised (expert) experience in oral surgery were collected.

**Table 1:** Face and content validity questionnaire used for the face and content validation of the perfusion-based mandibular model by the participants

	Strongly disagree (1)	Disagree (2)	Neutral (3)	Agree(4)	Strongly agree (5)
*Face validity*					
1. Visual appearance of bone tissue is realistic	1	2	3	4	5
2. Blood flow in the bone tissue (without defect) is realistic	1	2	3	4	5
3. Bleeding from bone defect is realistic	1	2	3	4	5
4. Colour of the blood is realistic	1	2	3	4	5
5. Viscosity of the blood is realistic	1	2	3	4	5
6. Resistance of the bone during drilling is realistic	1	2	3	4	5
7. Temperature of the tissues is realistic	1	2	3	4	5
*Task specific content validity*					
8. Filling of the bone defect with bone substitute is realistic	1	2	3	4	5
9. Application of the membrane over the bone defect is realistic	1	2	3	4	5
*Global content validity*					
10. This model would help to improve skills in handling the barrier membrane	1	2	3	4	5
11. This model would help to test the application of (adhesive) barrier membranes	1	2	3	4	5
12. This model replicates actual barrier membrane application	1	2	3	4	5

### Data analysis

Questions were categorized into two types of validity: face validity which refers to the “extent to which items used in a procedure appear superficially to sample that which is to be measured” or content validity which is described as “the extent to which the items of a procedure are in fact a representative sample of that which is to be measured” [10]. Validation data assessed on a 5-point Likert scale was presented as mean value ± standard deviation. For each question regarding validation, the mean rank of the two groups of participants were compared with a Mann–Whitney U test. Statistical analyses were performed with SPSS^®^ Statistics software (version 25, IBM SPSS Inc., Chicago, IL, USA). Statistical significance was considered when *p* < 0.05.

## Results

### Mandibular model

In eight of nine perfusion experiments, cannulation and connection of the porcine head to the perfusion system was uneventful. One experiment was stopped prematurely due to inability to cannulate the common carotid arteries that were cut too short. The model including activated clotting time (ACT) and haemodynamic parameters was stable for at least three hours. This also included (stable and consistent) bleedings from soft tissue incisions and bone tissue defects, indicating a well-distributed blood circulation of the mandibular tissues**.** In two experiments, blood flow was deliberately increased after the start of the experiment, which was done to improve the realism of the bleeding as judged by the researcher and participant.

### Study participants

Ten dentists, one OMF surgeon (expert group) and seven dental trainees (trainee group) participated in the study. Mean experience was 19.4 ± 16.9 years and 4.0 ± 1.7 years for the expert and trainee group, respectively. None of the participants had previous experience with in vivo or perfusion-based *ex vivo* models.

### Face validity

Participants gave a high score for the face validity of the model (mean of 3.9 ± 1.0, Table 2). Two-third of the participants assessed the defect bleedings as realistic, half of them scored the blood flow in the mandibular tissue as realistic. Ninety-four percent of the participants scored the colour of the blood as (highly) realistic while two-third of the participants agreed or strongly agreed on the question regarding the realism of the viscosity of the blood. However, some participants observed that hardly any coagulum was present in the defects and on instruments compared with a clinical situation.

**Table 2:** Trainees’ and experts’ rating of the perfusion-based mandibular model

	Question	Strongly disagree (1)		Disagree (2)		Neutral (3)		Agree (4)		Strongly agree (5)		*p*-value
		Trainees (%)	Experts (%)	Trainees (%)	Experts (%)	Trainees (%)	Experts (%)	Trainees (%)	Experts (%)	Trainees (%)	Experts (%)	
1	Visual appearance of bone tissue is realistic	0	0	0	0	0	0	4 (22.2)	3 (16.7)	3 (16.7)	8 (44.4)	0.218
2	Blood flow in the bone tissue (without defect) is realistic	0	0	1 (5.6)	3 (16.7)	3 (16.7)	2 (11.1)	2 (11.1)	4 (22.2)	1 (5.6)	2 (11.1)	0.925
3	Bleeding from bone defect is realistic	0	0	2 (11.1)	2 (11.1)	0	2 (11.1)	5 (27.8)	4 (22.2)	0	3 (16.7)	0.526
4	Colour of the blood is realistic	0	0	0	1 (5.6)	0	0	2 (11.1)	4 (22.2)	5 (27.8)	6 (33.3)	0.429
5	Viscosity of the blood is realistic	0	0	0	2 (11.1)	1 (5.6)	3 (16.7)	5 (27.8)	5 (27.8)	1 (5.6)	1 (5.6)	0.193
6	Resistance of the bone during drilling is realistic	0	0	0	0	2 (11.1)	0	2 (11.1)	8 (44.4)	3 (16.7)	3 (16.7)	0.839
7	Temperature of the tissues is realistic	1 (5.6)	0	1 (5.6)	2 (11.1)	1 (5.6)	3 (16.7)	2 (11.1)	6 (33.3)	2 (11.1)	0	0.702
8	Filling of the bone defect with bone substitute is realistic	0	0	0	0	2 (11.1)	0	3 (16.7)	6 (33.3	2 (11.1)	5 (27.8)	0.210
9	Application of the membrane over the bone defect is realistic	0	0	1 (5.6)	0	2 (11.1)	0	3 (16.7)	7 (38.9)	1 (5.6)	4 (22.2)	0.056
10	This model would help to improve skills in handling the barrier membrane	0	0	0	1 (5.6)	0	0	5 (27.8)	4 (22.2)	2 (11.1)	6 (33.3)	0.445
11	This model would help to test the application of (adhesive) barrier membranes	0	0	0	0	1 (5.6)	0	5 (27.8)	8 (44.4)	1 (5.6)	3 (16.7)	0.298
12	This model replicates actual barrier membrane application	0	1 (5.6)	1 (5.6)	0	2 (11.1)	1 (5.6)	4 (22.2)	6 (33.3)	0	3 (16.7)	0.147

Values are indicated as number of participants (n) and percentage of total participants (%) per question. *p*-value shows the significance of the comparison between the total mean ranks from experts versus trainees

All participants agreed that the visual appearance of the bone tissue was (highly) realistic and the feeling of drilling into bone was considered representative of the clinical situation by 88.9% of the participants. One participant suggested to use this model for common surgical skills training since many students experience difficulties with the manoeuvre of periosteum elevation in clinical practice. In 55.6% of the cases, the model was positively evaluated for tissue temperature, however 22.3% of the participants felt that the skin temperature was too low (16.7%) or too high (5.6%).

### Content validity

The model scored high on content validity (4.1 ± 0.8, Table 2). According to the participants, no major tasks or items on application of barrier membranes were lacking. The task-specific questions regarding filling the bone defect and the application of a barrier membrane, which were actions for product assessment for which the model was developed, were considered as (highly) realistic by 88.9% and 83.3% of the participants. No questions were included on periosteum elevation, nevertheless participants, especially students and young professionals, indicated that this was a highly realistic task in the model. The vast majority of the participants (94.4%) agreed or strongly agreed on the question that the model helps to improve skills in handling the barrier membrane. The participant who did not agree indicated that ‘several hurdles that are normally present in intraoral barrier membrane application were not present in the model, including limited space and sight, and surrounding tooth structures’. Although this question was not included in the questionnaire, the majority of the participants were positive about using the model as a training model for dental- and maxillofacial trainees for skills training with a blood perfused model. It was mentioned by one trainee that this model is ‘more realistic compared to the models that I use now to practice surgical procedures’. Participants mentioned different procedures that can be practiced for research or clinical practice, including periodontal, sinus restoration and oral implantation interventions.

Almost all participants (94.4%) did agree that this model would help to test the application of (adhesive) barrier membranes. Opinions were mixed on the question ‘This model reflects actual barrier membrane application’, however 72.2% of participants agreed on this question.

### Trainee versus expert opinions

**Fig. 3:**
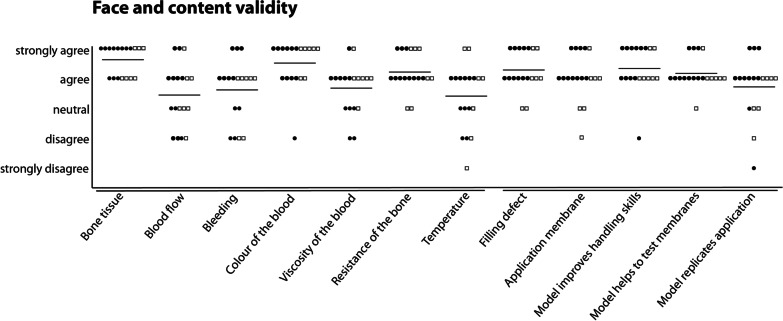
Scatter plot of face and content validity of the perfusion-based mandibular model. Every dot represent the answer of one participant, black dots are experts and white rectangles are trainees. Lines represent median

No difference was found between overall face validity scores of experts and trainees (both 3.9 ± 1.0, Fig. 3), nor in the individual face validity items. Highest, but non-significant difference was found regarding the viscosity of the blood item, which was more positively scored by trainees (mean score of 4.0 ± 0.6) compared with experts (mean score of 3.5 ± 0.9).

Experts were slightly more positive (4.2 ± 0.8) compared with trainees (3.9 ± 0.8) about overall content validity. For all individual content validity items, experts assessed the model more positively compared with trainees. This was particularly true for the question ‘Application of the membrane over the bone defect is realistic’, which was more often evaluated as realistic by experts with a mean score of 4.4 ± 0.5 compared to trainees (3.6 ± 1.0, *p* = 0.056).

## Discussion

We validated a novel clinically relevant perfusion-based mandibular pig model for barrier membrane research that has merit to identify initial performance and handling characteristics of laboratory prototypes. The model reached high scores for both face validity and content validity, which were comparable between trainees and experts on most aspects. Demonstrating these validities holds promise for reducing and replacing living animals for biomaterial research and for related surgical training purposes.

Since non-heparinized blood affected functioning of the pump and oxygenator of the model, and previous attempts to neutralize the heparin with protamine failed, the donor blood had to be heparinized. The subsequent changes in blood viscosity and clot formation associated with bleeding [11] could have affected results on items regarding the appearance and the viscosity of the blood. Although validity scores were relatively high in both groups, most participants commented on the lack of blood clots in the operative field as less realistic in this model. From high task specific scores, the heparinized blood did not seem to jeopardize the conduct of the procedure including filling the bone defect and placement of the membrane. One could argue that the inevitable use of heparin makes this model more suitable to mimic complex clinical conditions of coagulation disorders or anti-coagulant drug use, increasingly encountered in a dental or maxillofacial surgery practice [12].

The questionnaire used to validate the model was adapted from a previously published and validated questionnaire regarding validity of a new model for surgical training [9]. No questionnaires on face and/or content validity other than training models were available in literature. In addition, no validity questionnaires on research or product assessment were found. Although all questions used in this study were tested on reliability and uniform text interpretation, the questionnaire was not validated for the use in maxillofacial and dental research and product assessment. This may have affected the consistency of the questionnaire and reliability of the results, so conclusions must be drawn with caution [13, 14].

We invited participants of different experience levels to evaluate the robustness of the validity and the experience range of applying this model. Face validity was rated similar in both groups, content was found more valid by experts on items related to biomaterial handling and placement. This difference can be explained by the lack of experience of trainees performing the operation in patients independently themselves. A better understanding of the key elements of a procedure comes from experience, and this can and needs to be trained in a simulation setting [15]. Including trainees in this study also has the advantage of procedural skills training that were mentioned for this model. This shows that the model is not only usable for research but also for educational purposes.

The model was originally developed to assess bone adhesive barrier membranes for dentistry and maxillofacial surgery. Therefore, the focus of the questionnaire was on tissues and tasks involved in the application of the bone adhesive membranes in an in vivo setting. Questions addressing bone tissue and blood appearance were all very positively rated by both experts and trainees while more mixed opinions were present in questions regarding bleeding and blood flow. Since the vasculature in bone tissue is buried and the mandible has relatively little blood flow [16], assessing the perfusion quantity before the bone tissue is uncovered is rather difficult. This may have resulted in differences in rating blood flow and bleeding at the start compared to the continuation of the experiment, particularly in those situations where the perfusion variables were adjusted.

To the best of our knowledge this is the first study of an *ex vivo* mandibular model development and validation for evaluation of new biomaterials. Most studies on the development of new models focus on acquiring or improving surgical skills [17–21]. A few studies addressed *ex vivo* model for drug passaging [22] or organ transplant research [23]. However, most models are less realistic and suitable for research purposes compared with our model due to stiffness, a lower (room) temperature of tissues, and absence of bleeding from surgical defects. In addition our model holds promise as training tool for dental and maxillofacial professionals to practice complex surgical procedures or for simulation training by students and experts. Considering model validity for simulation training, our study results compare favourably with those reported by Shen et al. (2017) who validated a perfusion-based human cadaveric model (face validity, 4.82 ± 0.41; content validity, 4.88 ± 0.33) for endoscopic endonasal sinus and skull base surgical procedures [20]. Similar full body human cadaveric models were evaluated by Danion et al. [24] and Buchanan et al. [25] as training specimen for a variety of surgical procedures. However, these studies made use of full cadaveric human bodies, which are laborious, expensive and associated with ethical concerns. With a simpler pulsatile organ perfusion (POP trainer, OPTIMIST Ltd, Innsbruck, Austria) model for surgical skills training, validity scores (realism, 3.8 ± 0.9; usefulness, 4.6 ± 0.9; range 1–5) were comparable to our scores [26]. However, the POP trainer was rated significantly higher by novices compared to experts for its overall usefulness, which was the opposite in our study. This contrast may be explained by the less complex tasks performed in the POP trainer.

Common training models available for dental researchers and trainees include human and animal cadavers, industrially manufactured typodont models and customized 3D printed models [27–29]. These generally lack a blood circulation and some have limited resources due to ethical and logistical concerns. Although 3D printed models can be customized for different treatments and diseases, animal and cadaveric dental models composed of multiple tissues are generally evaluated more positively [28]. The benefit of the composed tissue models as the perfused-based mandibular model can be found in the versatility of the models regarding surgical skill practice (*e.g.* tooth extraction, biopsy, sutures, gingival procedures and sinus floor elevations [30, 31], as well as practising administration of local anaesthetics [32]). This makes it an interesting model for a variety of disciplines *e.g.* researchers, dentists, OMF surgeons, oral hygienists and dental assistants.

A limitation of the novel *ex vivo* perfusion-based model is that it is laborious, which includes building, disassembling and cleaning the set-up. A smaller and portable perfusion design would be more convenient for future studies and will reduce the experimental time. Furthermore, the model is dependent on slaughterhouse resources, the availability of equipment and facility to work with and transport animal materials. A fresh cadaveric head and a short transportation time to the laboratory is strongly recommended since this will limit blood clots in the porcine head, however this logistic challenge might complicate the general usability of the model. The relatively low number of participants per groups (experts vs trainees) may be considered a limitation for the comparison outcomes. However, the comparison between experts and trainees was a secondary objective of the study. We do not expect that a larger number of participants would have affected results regarding the face and content validity of the model.”

The development and validation of the novel perfusion-based mandibular pig model contributes to a new generation of *ex vivo* models with a more clinical relevant output. The model can be used for a number of purposes in dentistry and maxillofacial surgery, including the assessment of new dental haemostats, oral implants and surgical procedures in presence of challenging circumstances with heparinized full blood. In addition, new haemostats can be assessed since there is proof that the blood values of the model are stable for 3 + hours [6]. Based on early findings in our laboratory, the novel model might replace a significant number of living animals generally used in early stages of product development since the animals were slaughtered for human consumption. The use of new clinically relevant *ex vivo* models must be encouraged by research institutes, industry and local animal ethics committees to further expanding the applications. However, further specific validation of the model for new procedures is needed. In addition, it is recommended to develop a model that is more portable and requires only a limited amount of heparin in the blood.

## Conclusion

This study established face and content validity of a novel perfusion based mandibular model. The model holds promise to assess bone adhesive barrier membrane prototype selection based on feasibility and early efficacy, and may replace a first series of animal studies.

## Data Availability

The data that support the findings of this study are available from the corresponding author (MvE) upon reasonable request.

## Abbreviations

OMF: Oral maxillofacial
ACT: Activated clotting time
